# Correcting for Strong Eddy Current Induced B0 Modulation Enables Two-Spoke RF Pulse Design with Parallel Transmission: Demonstration at 9.4T in the Human Brain

**DOI:** 10.1371/journal.pone.0078078

**Published:** 2013-10-21

**Authors:** Xiaoping Wu, Gregor Adriany, Kamil Ugurbil, Pierre-Francois Van de Moortele

**Affiliations:** Center for Magnetic Resonance Research, Department of Radiology, University of Minnesota Medical School, Minneapolis, Minnesota, United States of America; University of Ulm, Germany

## Abstract

Successful implementation of homogeneous slice-selective RF excitation in the human brain at 9.4T using 16-channel parallel transmission (pTX) is demonstrated. A novel three-step pulse design method incorporating fast real-time measurement of eddy current induced B0 variations as well as correction of resulting phase errors during excitation is described. To demonstrate the utility of the proposed method, phantom and *in-vivo* experiments targeting a uniform excitation in an axial slice were conducted using two-spoke pTX pulses. Even with the pre-emphasis activated, eddy current induced B0 variations with peak-to-peak values greater than 4 kHz were observed on our system during the rapid switches of slice selective gradients. This large B0 variation, when not corrected, resulted in drastically degraded excitation fidelity with the coefficient of variation (CV) of the flip angle calculated for the region of interest being large (∼12% in the phantom and ∼35% in the brain). By comparison, excitation fidelity was effectively restored, and satisfactory flip angle uniformity was achieved when using the proposed method, with the CV value reduced to ∼3% in the phantom and ∼8% in the brain. Additionally, experimental results were in good agreement with the numerical predictions obtained from Bloch simulations. Slice-selective flip angle homogenization in the human brain at 9.4T using 16-channel 3D spoke pTX pulses is achievable despite of large eddy current induced excitation phase errors; correcting for the latter was critical in this success.

## Introduction

Parallel transmission (pTX) in the context of magnetic resonance imaging [1]–[4] consists of simultaneously playing different RF pulse shapes on independent transmit (Tx) channels. It is known that by designing multidimensional (multi-D) spatially selective pTX RF pulses, in concert with time varying encoding gradients, one can mitigate the impact of Tx B1 (B1+) nonuniformities [5]–[7] as well as achieve arbitrarily shaped RF excitation profiles [3], [8], [9]. Such techniques are especially relevant at ultra-high magnetic fields (i.e., 7T and beyond) where B1+ profiles in human anatomical targets are notoriously heterogeneous. However, multi-D RF pulses are highly sensitive to deviations in excitation k-space trajectory arising from system imperfections such as gradient amplifier nonlinearities, time delays or residual eddy currents [10], [11].

We have demonstrated *in-vivo* on a 9.4 T human system large improvement in 2D pTX excitation fidelity by including susceptibility induced main field inhomogeneities (ΔB_0_) maps and actual k-space trajectories, measured on the fly, in pTX RF pulse design involving encoding gradients along x- and y-axes [10]. However, when expanding this approach towards slice-selective 3D spoke pTX RF pulse design to achieve homogeneous excitation within axial slices on the same scanner, significant residual excitation errors were observed, even though in this case *measured* and *nominal* k-space trajectories were virtually identical [12], [13]. We hypothesized that this was due to eddy current induced zero-order B0 modulations, resulting from slice-selective z-axis encoding gradient switches, that are not described in a conventional k-space trajectory representation limited to linear spatially encoding gradients [13]. In the current work we detail the successful implementation of 3D pTX in the human brain at 9.4T with drastically improved excitation fidelity when correcting for eddy current induced B0 variations. A three-step pulse design is proposed:

1. *In-vivo* measurement of eddy current induced B0 modulation, B1+ and ΔB_0_ maps.
2. Initial 3D pTX pulse design based on B1+ and static ΔB_0_ maps.
3. Final 3D pTX pulse design with correction of eddy current induced B0 modulation.

## Materials and Methods

### Ethics Statement

The human experiments performed in the present study were approved by the local institutional review board of the University of Minnesota. Healthy volunteers who signed a written consent form were recruited and received compensation for their participation.

### General setting and hardware

All experiments were conducted on a 9.4T, 65-cm-diameter inner bore MR human scanner (Magnex, Oxford, UK) driven by a DirectDrive console (Agilent, Santa Clara, CA). The scanner was equipped with a head gradient coil (Magnex, Oxford, UK) with an inner diameter of 40 cm, powered by a Siemens gradient amplifier (Siemens, Erlangen, Germany) allowing a maximum gradient strength of 48 mT/m and a maximum slew rate of 240 T/m/s. Prior to the current study, the calibration of pre-emphasis currents to counteract gradient induced eddy currents had been performed on the system following standard manufacturer procedures [14]. Note that the compensation of eddy current induced B0 variations is obtained by applying a time varying current on a physical B0 coil (capable of generating a frequency modulation up to about 4 kHz) rather than by modulating in real time the carrier RF frequency. The DirectDrive console was equipped with 16 independent Tx channels, each powered by a 500W RF amplifier (CPC, NY, USA). As previously described [10], [15], [16], a home-built multi-channel RF power monitoring unit continuously measured the output power of each RF amplifier to ensure compliance with safety guidelines on maximum specific absorption rate. A 16-channel Transmit/Receive open-faced head array [17], consisting of separable upper and lower coil formers each with eight resonance elements, was used for both RF transmission and reception. When combined, the upper and lower formers resulted in an elliptical shape in the transverse plane (20 and 24 cm long in the left-right and anterior-posterior directions, respectively). Each array element was built using a 12 mm wide copper strip conductor and a 42 mm wide RF ground plane, with 12 mm thick Teflon dielectric in between. All MR signals were simultaneously acquired on 16 independent receive channels. Phantom studies were performed prior to the *in-vivo* experiments to test and optimize the imaging protocols, using a spherical saline phantom (99 mM NaCl, 16 cm in diameter) doped with copper sulfate (T1 ∼ 200 ms) as in Ref. [10] where the relatively short T1 value allowed for fast acquisition without having to use a long repetition time to attain sufficient longitudinal magnetization relaxation. In-vivo experiments were obtained in three human subjects using the same acquisition protocol.

### Gradient and Eddy Current Characterization

The present study is focused on pTX pulse design aiming at achieving homogeneous excitation within axial slices using multiple slice-selective RF sub-pulses with spatially encoding gradient blips along the x and y axes inserted between the sub-pulses. Slice-selective spoke gradients were always applied along the *Z* direction, and the eddy current induced B0 modulations were measured using a gradient characterization approach previously described [18]. The pulse sequence developed for data acquisition comprised a conventional slice-selective RF excitation along the *Z* direction, immediately followed by the application of the spoke gradient during which MR signals are sampled (with neither phase encoding nor readout gradients). The phase evolution
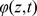
of the signal due to the spoke gradient within a slice positioned at a distance *z* from the gradient isocenter can be described as:
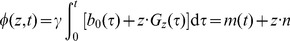
(1)


with
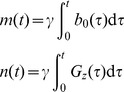



Here
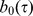
is the eddy current induced B0 variations (i.e., zero order eddy current which is spatially invariant),
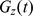
the linear gradient including corresponding first order eddy current terms, and
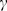
the proton gyromagnetic ratio.

Similar to Ref. [18], 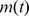
and
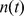
were first obtained by fitting
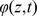
of a multi-slice dataset to the linear model of Eq. 1 with respect to the varying slice positions *z* for each time point *t*, and were then used to calculate
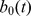
and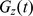, respectively. Phase accumulations arising from other sources such as main field inhomogeneities (ΔB0) and slice selective gradient used for slice excitation (not the measured spoke gradient) were also obtained with the spoke gradient turned off and were removed from the signal phases so as to obtain phase evolutions purely due to the spoke gradient. In both phantom and human experiments, the slice selective RF excitation was achieved using a low-bandwidth RF pulse (8 ms in length), and MR signals, sampled at a temporal resolution of 4 μs, were obtained in an interleaved manner in three slices (slice thickness  =  1.5 mm) positioned at *z*  =  –10, 0 and 10 mm, respectively. To improve SNR, a multi-slice dataset averaged over 32 acquisitions was taken to calculate the eddy current induced B0 shifts. The total acquisition was obtained within 15.4 s with TR/TE  =  240/10.8 ms.

### B1 and ΔB0 Field Mapping

**Figure 1:**
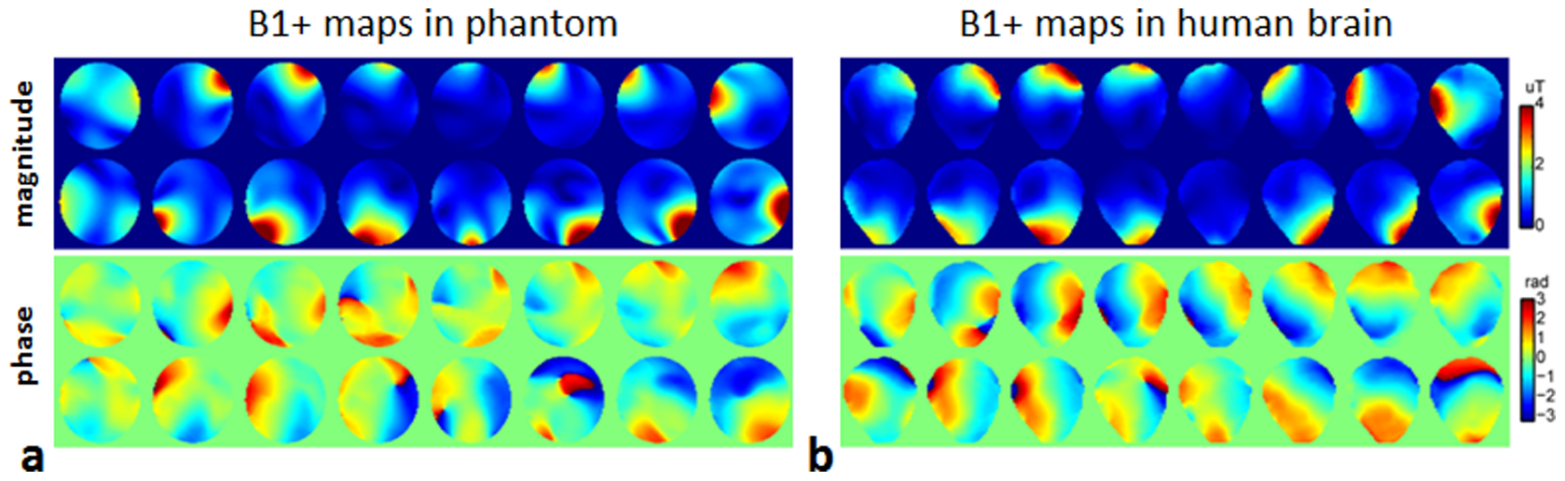
Transmit B1 maps measured for the 16-channel transceiver in the phantom (a) and in the human brain (b). The B1+ magnitude and relative phase maps are shown in the top and bottom panels, respectively.

Transmit and receive complex B1 maps as well as ΔB0 maps were obtained as previously described [10]. Briefly, complex-valued B1+ maps for all 16 channels were obtained with a fast hybrid multi-channel B1+ mapping technique where one absolute flip angle map is merged with 16 relative B1+ maps to generate 16 absolute |B1_k_+| maps (k = 1,2,…,16), exploiting the knowledge of complex interferences between channels derived from the relative B1+ maps [19]. The 16 relative B1+ maps were derived from a series of small tip angle gradient echo (GRE) images with one channel transmitting at a time, acquired with TR/TE  =  100/2.3 ms, slice thickness  =  5 mm, matrix size  =  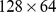, acquisition time  =  115 s, and FOV  =  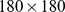 mm^2^ for the phantom and 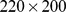 mm^2^ for the human head. The absolute B1+ magnitude map was obtained, with all channels transmitting together, using the actual flip angle imaging (AFI) method [20] with TR1/TR2/TE  =  25/125/2.6 ms, slice thickness  =  9 mm, matrix size  =  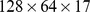, acquisition time  =  165 s, and FOV  =  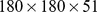 mm^3^ for the phantom and FOV  =  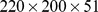 mm^3^ for the human head. Fig. 1 displays the 16 complex B1+ maps measured in the phantom and in the human brain. An additional small tip angle GRE image was obtained using the same RF pulse as in the AFI and was divided by the sine of the AFI flip angle map to estimate the receive B1 magnitude (|B1-|) multiplied by proton density (*ρ*). Note that in such estimation one would expect residual T1-related signal variations in the brain since a relatively short TR (100 ms) was used. ΔB0 maps were derived from two GRE images (TE1/TE2  =  5/5.5 ms) and were incorporated into the pulse design to avoid distortions of excitation pattern due to susceptibility induced off-resonance. All field maps were down-sampled to match the spatial resolution used in the subsequent pulse design.

### Initial RF Pulse Design

3D pTX RF pulses were designed to achieve uniform excitation within an axial slice, using the nominal two-spoke excitation k-space trajectories. These RF pulses consisted of two Gaussian-shaped sub-pulses (1 ms in length, time-bandwidth-product  =  2), each applied during the plateau of a trapezoidal slice-selective spoke gradient (*G*_z_) with maximum amplitude of 16.5 mT/m and maximum slew rate of 150 T/m/s, in order to obtain a slice thickness of 5 mm.

Complex coefficients (i.e., RF magnitude and phase) for individual channels and individual sub-pulses were calculated with the magnitude least squares optimization [21]:
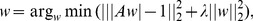
(2)


where
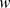
is a 32-element (16 × 2  =  32) vector of complex-valued coefficients, *A* is a complex-valued matrix incorporating the B1+ sensitivities of individual channels, modulated by the Fourier kernel corresponding to spoke placements in k-space as well as by the measured static ΔB0 maps, and
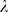
is a real-valued regularization parameter used to determine a tradeoff between excitation error and total RF power.

A uniform 10° excitation target was defined on a 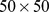 grid with in-plane isotropic spatial resolution of 3.6 mm for the phantom and of 4.4 mm for the human brain. Similar to Ref. [6], the spoke placement in the transverse k-space was constrained to be symmetric about the origin and was optimized over a grid defined by an array of rotation angles (from 0° to 170° with 10° increments) and an array of spatial frequencies (from 8 to 18 cm^−1^ with 2 cm^−1^ increments). After the spoke placement was determined, the regularization parameter 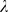 used for pulse calculation was chosen based on the L-curve criterion [3]. Only pixels inside the region of interest (ROI), i.e. within the phantom or brain tissues, were considered in the pulse design. All waveforms were defined with a dwell time of 4 μs, and the total pulse duration including the slice-selective gradient rewinder lobe was 3 ms.

### Corrected RF Pulse Design

In order to achieve satisfactory excitation quality, RF pulses were corrected for eddy current induced B0 variations arising from the application of the spoke gradient. The corrected pulse 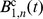 for the *n*-th channel was obtained by applying a phase modulation to the initially calculated pulse 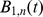 based on the eddy current measurement:
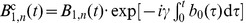
(3)


In practice, this spatially invariant phase modulation is equivalent to a time-varying modulation of the carrier RF frequency. Note that no trajectory correction (i.e. no linear term Gx, Gy or Gz) was incorporated in the proposed pulse design since we found no difference in excitation patterns when comparing the use of nominal and measured transverse gradient blips for pulse calculation in our initial investigation.

### Experimental verification

The excitation patterns for the initial and corrected pTX pulses were measured using 3D AFI, with TR1/TR2/TE  =  25/125/3.6 ms, matrix size  =  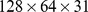, FOV  =  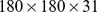 mm^3^ for the phantom and FOV  =  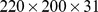 mm^3^ for the human head, nominal flip angle  =  50° in the phantom and 40° in the human brain. The coefficient of variation (CV), defined as the std/mean of flip angle values within the ROI, was calculated for quantitative comparison between different pulses. Additional GRE images were obtained, using a modified 3D GRE pulse sequence where the RF excitation module was replaced by the initial or the corrected pTX pulse, with nominal flip angle  =  10°, TR/TE  =  100/4 ms, matrix size  =  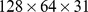, FOV  =  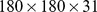 mm^3^ for the phantom and 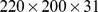 mm^3^ for the human head, acquisition time  =  199 s. These GRE images were further normalized by the estimated product
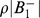
as described earlier in order to compare the excitation patterns.

### Simulations

Bloch simulations were conducted to calculate excitation patterns with initial and corrected pTX RF pulses. To reproduce as closely as possible the experimental conditions, these simulations included the measured eddy current induced B0 variations as well as the measured gradient, B1+ and ▵B0 maps. Excitation patterns were defined, as for the experimental settings, on a 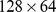 grid with a field of excitation of 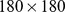 mm^2^ for the phantom and of 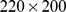 mm^2^ for the human head. All simulations assumed uniform longitudinal magnetization at equilibrium and ignored relaxation and diffusion effects. All computations, including RF pulse design and Bloch simulations, were performed in Matlab (MathWorks, Natick, MA, USA).

## Results

**Figure 2:**
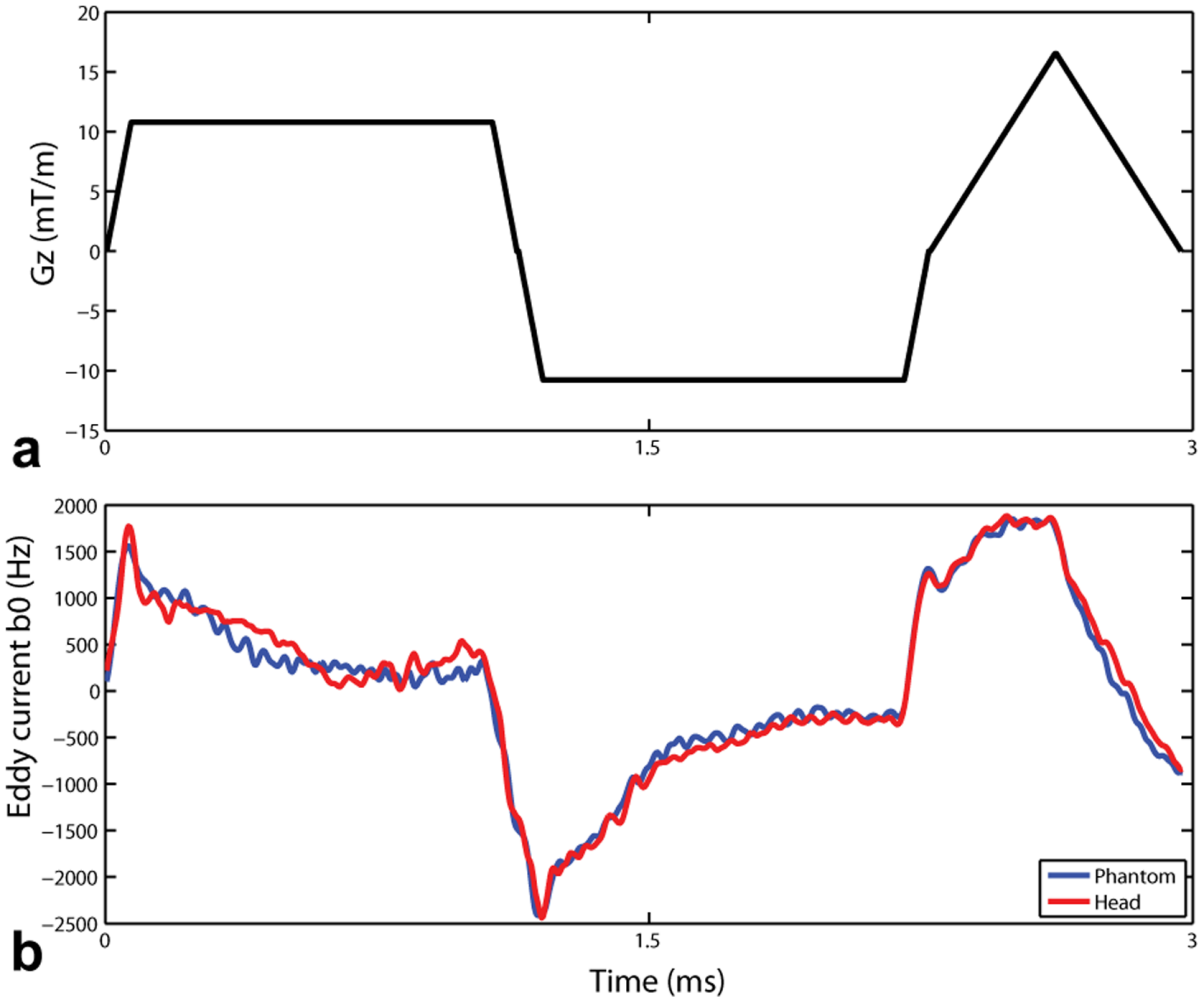
The 2-spoke slice selective gradient waveform (a) used in the pulse design and the resulting eddy current induced B0 shifts (b) measured in the phantom (blue curve) and in the human brain (red curve). Note that large eddy current induced B0 variations with about 4-emphasis eddy current compensation.

Large eddy current induced B0 variations were observed on our system, with peak-to-peak values greater than 4 kHz and with fairly short time constants (<1 ms) during the rapid switches of the 2-spoke slice selective gradients (Fig. 2). A comparison between the blue and red curves in Fig. 2b indicates a high consistency between phantom and human brain based measurements.

**Figure 3:**
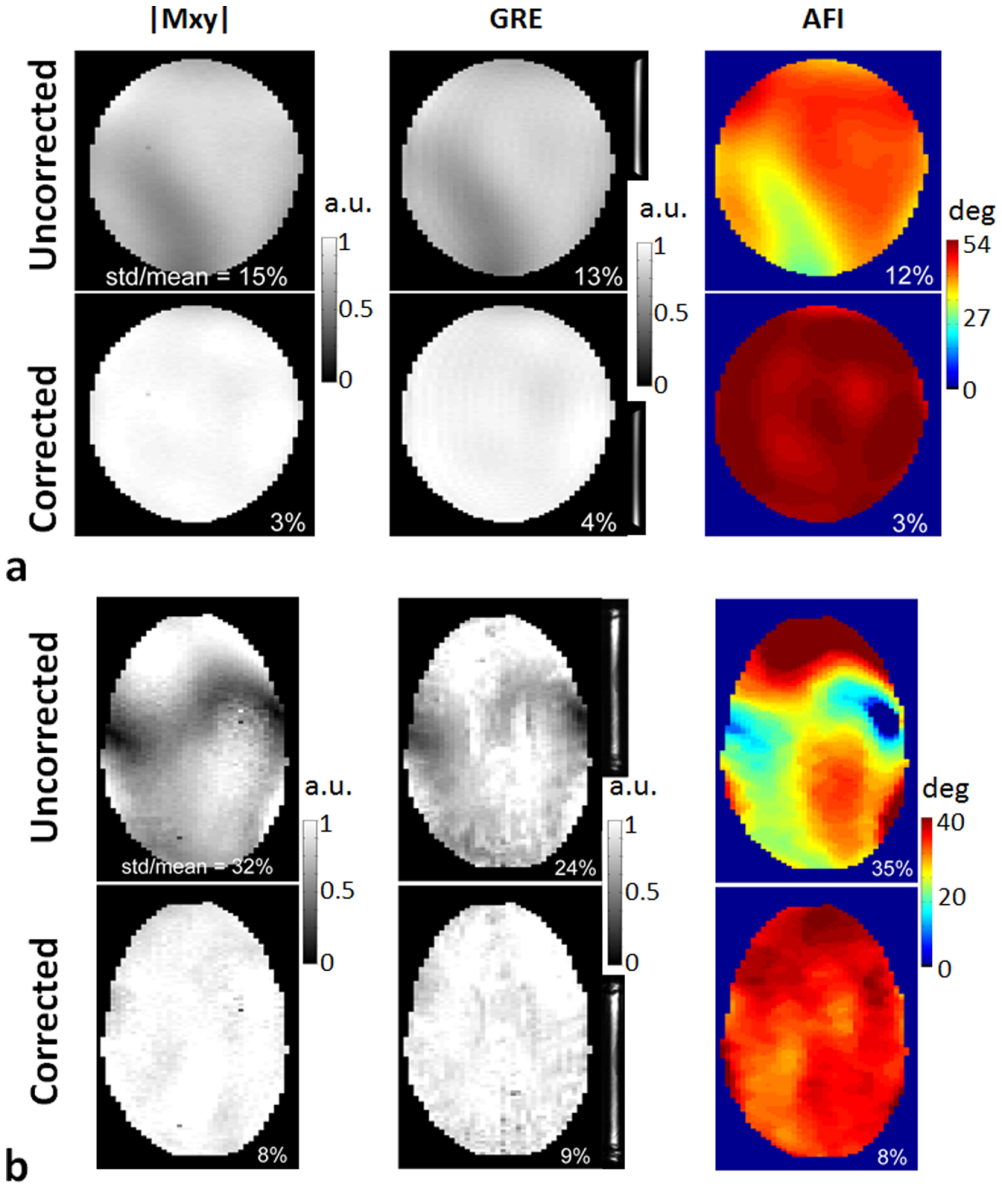
Uncorrected vs corrected 2-spoke pTX RF pulses in terms of excitation fidelity evaluated in the phantom (a) and in the human brain (b) at 9.4 T. The coefficient variation values (i.e., std/mean) for both uncorrected and corrected pulses quantifying the flip angle inhomogeneities were calculated based on the corresponding Bloch simulations (left column), 3D GRE images normalized with respect to receive B1 (middle column) and actual flip angle imaging (right column). A mid-sagittal slice of the original 3D GRE acquisition is also shown on the right of each normalized GRE image, to appreciate the intact slice selectivity of both the uncorrected and corrected RF pulses. A nominal flip angle of 10° was used in both Bloch simulation and 3D GRE acquisition, and a higher nominal flip angle of 50° for the phantom and 40° for the brain was employed in the flip angle mapping. Note that in both the phantom and the human brain signal voids due to eddy current induced B0 shifts observed with the uncorrected RF pulses were effectively restored with significantly improved excitation homogenization when using the corrected pulses. Additionally, good agreement was seen between numerical predictions and experiments.

When these B0 temporal modulations were not accounted for in the pulse design, the excitation pattern |Mxy|, as predicted by Bloch simulations, was drastically degraded for both phantom and human brain data, and the corresponding experimental data very closely reproduced these predicted distortions both in small flip angle GRE images and in measured flip angle maps (Fig. 3). By contrast, excitation homogeneity dramatically improved when eddy current induced B0 temporal modulations were corrected in pTX RF pulse design (Fig. 3). The slice selectivity remains unchanged between uncorrected and corrected pulses as seen in a mid-sagittal slice of the 3D GRE acquisition (Fig. 3, middle column). CV values in phantom data as measured by |Mxy| (via Bloch simulations), GRE image signal intensities, and flip angle maps decreased from 15%, 13% and 12% *without* correction for eddy current induced B0 shifts, down to 3%, 4% and 3% *with* correction, respectively. Corresponding CV values in the brain dropped from 32%, 24% and 35% down to 8%, 9% and 8%, respectively.

**Figure 4:**
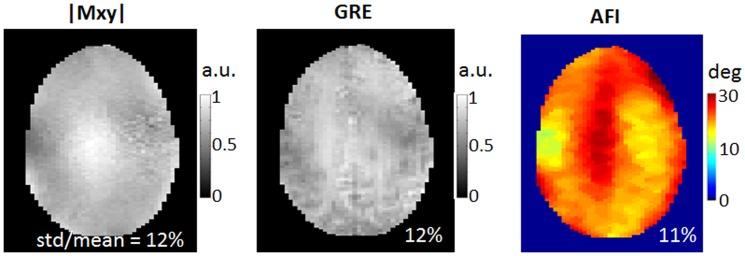
Excitation fidelity of static B1 shimming (i.e., single-spoke pTX pulse design) in the human brain at 9.4 T using eddy current B0 corrected single-spoke pTX pulses. The coefficient variation values (i.e., std/mean) were calculated based on the corresponding Bloch simulations (left column), 3D GRE images normalized with respect to receive B1 (middle column) and actual flip angle imaging (right column). A nominal flip angle of 10° was used in both Bloch simulation and 3D GRE acquisition, and a higher nominal flip angle of 23° was employed in the flip angle mapping. Note that B1 shimming was not as effective as 2-spoke pTX pulse design in creating uniform excitation.

Figure 4 displays the predicted excitation patterns |Mxy|, GRE acquisition and actual flip angle mapping in the human brain when using eddy current corrected single-spoke pTX pulses (i.e., static B1 shimming), obtained with imaging parameters similar to those for the 2-spoke pulse design. As can be seen, the CV numbers achieved with B1 shimming were larger than those with 2-spoke pulses, showing that B1 shimming is not as effective as the 2-spoke pulse design in B1+ homogenization.

## Discussion

In this study, we have demonstrated the successful implementation of 16-channel 3D slice-selective pTX RF excitation in the human brain at 9.4 T, counteracting large eddy current induced excitation errors in the pulse design. Importantly, our results show that very fast eddy current calibration can be obtained *in-vivo*, providing reliable results for excitation RF pulse design, suitable as a routine step in MR protocols. In the proposed approach an initial version of the pTX pulses is first calculated without eddy current compensation, based on the B1+ maps and taking into account the measured static ΔB0 maps. Eddy current induced temporal B0 modulations associated with the multi-spoke slice selective gradients, measured on the fly, were then used to correct the RF pulse by incorporating the corresponding phase variations. Experimental results acquired both in a phantom and in the human brain demonstrated that this compensation effectively mitigated signal loss and pattern distortion observed with the initial uncorrected RF pulses, resulting in satisfactory in-plane excitation homogeneity despite of severe eddy currents induced by the slice selective gradients.

Although pre-emphasis settings used to counteract gradient induced eddy currents were always active during this study, large amplitudes of B0 variations were still present during multi-spoke gradient waveforms with slice selection set along the z axis, possibly corresponding to the limits of the pre-emphasis hardware resources on our system to address high amplitude, short time constant B0 eddy currents. It is remarkable that transient B0 variations as large as 4 kHz could be addressed by correcting the pTX RF pulse, yielding satisfactory excitation fidelity. Recently, Schneider et al. [11] proposed an MRI based technique to map local phase accumulations of the transverse magnetization as a result of gradient applications and susceptibility effects during spatially selective RF excitations; this technique, however, is complex to implement and requires a relatively long calibration. By contrast, the calibration method used in the current study is easy to implement and requires very short acquisition time while providing reliable results.

It is interesting to note that here we did not observe any significant impact of eddy current induced gradient distortions (in Gx, Gy or Gz) on the excitation quality, whereas we had reported in a previous study, on the same system, major deteriorating impact of k-space deviations when using 2D Transmit SENSE RF pulses [10]. This can be explained by the fact that the two gradient blips used for the 2-spoke pulse design in the present study are very small in amplitude (not exceeding 4 mT/m) and very short in time (∼ 350 µs), yielding negligible excitation phase errors due to gradient imperfection. By contrast, the 2D spiral gradients that were applied during 2D Transmit SENSE pulses in the former study were 5-fold stronger in maximum amplitude and 7-fold longer in duration, yielding large k-space errors due to gradient distortions that could not be ignored. On the other hand, we observed in the present study large residual eddy current induced B0 shifts after switching the z-gradient coil, even with system pre-emphasis activated, whereas we did not observe such large B0 variations when using 2D Transmit SENSE pulses where the x- and y-gradient coils were highly solicited. We hypothesize that these differences in response between, on the one hand, the x-gradient and y-gradient coils, and, on the other hand, the z-gradient coil, are the result of inherent hardware characteristics of the gradient coil and of its interaction with the magnet.

Note that although demonstrated at the high field strength of 9.4 T, the proposed technique would also be applicable at lower field strengths (such as 3 T), whenever residual eddy current induced B0 shifts would be large enough to not be ignored during multi-spoke RF pulse design. Likewise, the proposed technique aims at providing satisfactory excitation pattern by addressing eddy current induced B0 modulations during the RF excitation and therefore can be used for the excitation module (up to 90° flip angles) in any conventional sequence when such correction is necessary. Further investigations, however, will be needed to see how such phase errors could be overcome when large flip angles are used, such as in pTx RF refocusing pulses in diffusion weighted imaging where residual eddy current induced B0 shifts may also be present following the application of diffusion gradients.

Although the focus of the current study is not on comparing different pTX pulse design strategies, it can be noticed that using only two spokes with 16 Tx channels already provides fairly homogeneous excitation profile through a 2D slice in the human brain, outperforming static B1 shimming results (Fig. 4). Two-spoke pTX RF pulses can still be fairly short, with limited sensitivity to *T*_2_ effects during RF excitation while preserving reasonably TE values, and therefore are expected to be suitable for a large array of imaging applications at 9.4T.

## Conclusion

We have demonstrated the successful implementation of 16-channel 3D parallel transmission in the human brain at 9.4T, with a multi-step RF pulse design strategy involving fast real-time calibration, capable of correcting for large eddy current induced excitation phase errors in addition to compensating for susceptibility induced off-resonance effects.
